# Ceritinib efficacy in SMARCA4-deficient NSCLC harboring novel CTNND2 ALK/EML4-ALK fusion: case report

**DOI:** 10.3389/fonc.2026.1680523

**Published:** 2026-01-28

**Authors:** Zhigang Fu, Gengda Huang, Jian Xie, Li Luo, Qinqin Ren, Hong He, Yuan Wang, Jiexia Zhang

**Affiliations:** 1 The Seventh Affiliated Hospital of Southern Medical University, Foshan, China; 2 Guangzhou Institute of Respiratory Disease, State Key Laboratory of Respiratory Disease, Department of Respiration, The First Affiliated Hospital of Guangzhou Medical University, Guangzhou, China; 3 Department of Radiation Oncology, The First Affiliated Hospital,Sun Yat-sen University, Guangzhou, China; 4 Department of Pulmonary and Critial Care Medicine of Jiangbei Guangzhou Medical University, Guangzhou, Guangdong, China; 5 Department of Pulmonary and Critial Care Medicine of Jiangbei Campus,The First Affiliated Hospital of Army Medical University, Chongqing, China

**Keywords:** ALK double-fusion, ceritinib, CTNND2-ALK, NSCLC, SMARCA4-dNSCLC

## Abstract

SMARCA4-deficient non-small cell lung cancer (SMARCA4-dNSCLC) is an aggressive malignancy with poor prognosis, rarely harboring EGFR, ALK, or ROS1 alterations. We report an advanced SMARCA4-dNSCLC case with brain metastasis exhibiting a novel CTNND2-ALK/EML4-ALK double-fusion. Following platinum-based chemotherapy and brain radiotherapy, next-generation sequencing identified the dual fusions (abundances: 2.6% and 5.2%), confirmed by ALK protein expression. The patient subsequently received ceritinib (750 mg/day). After 3 months, targeted lesions regressed significantly, and progression-free survival exceeded 24 months with ongoing response. This demonstrates efficacy of the ALK inhibitor ceritinib in ALK-rearranged SMARCA4-dNSCLC and underscores the clinical value of genomic-guided therapy.

## Introduction

The switch/sucrose non-fermenting (SWI/SNF) complex acts as a tumor suppressor across diverse biological processes (1), with subunit aberrations occurring in ∼20% of human cancers (2). SMARCA4, located at 19q13, encodes BRG1-an essential ATPase subunit of this complex. Thoracic SMARCA4-deficient tumors are classified as either SMARCA4-deficient non-small cell lung cancers (SMARCA4-dNSCLCs) or SMARCA4-deficient undifferentiated thoracic tumors (SMARCA4-UTs). The 2021 WHO classification designates SMARCA4-UT as a distinct entity due to its unique histologic, immunohistochemical, and clinical features (3).

BRG1 protein loss, with or without SMARCA4 mutations, affects 5-10% of NSCLCs, predominantly in male heavy smokers (4). Unlike conventional NSCLC, SMARCA4-dNSCLCs and SMARCA4-UT rarely harbor actionable drivers (e.g., EGFR, ALK, or ROS1) (5, 6), lack standard therapies, and confer poorer prognoses (5). While single EML4-ALK fusions occur in ~5% of NSCLCs, double ALK fusions involving EML4-ALK plus another partner (e.g., CTNND2) are exceptionally rare and may exhibit distinct drug sensitivity profiles (7).

Here, we report the first documented case of advanced SMARCA4-dNSCLC with a novel CTNND2-ALK/EML4-ALK double-fusion, demonstrating sustained response to first-line ceritinib.

## Case presentation

**Figure 1:**
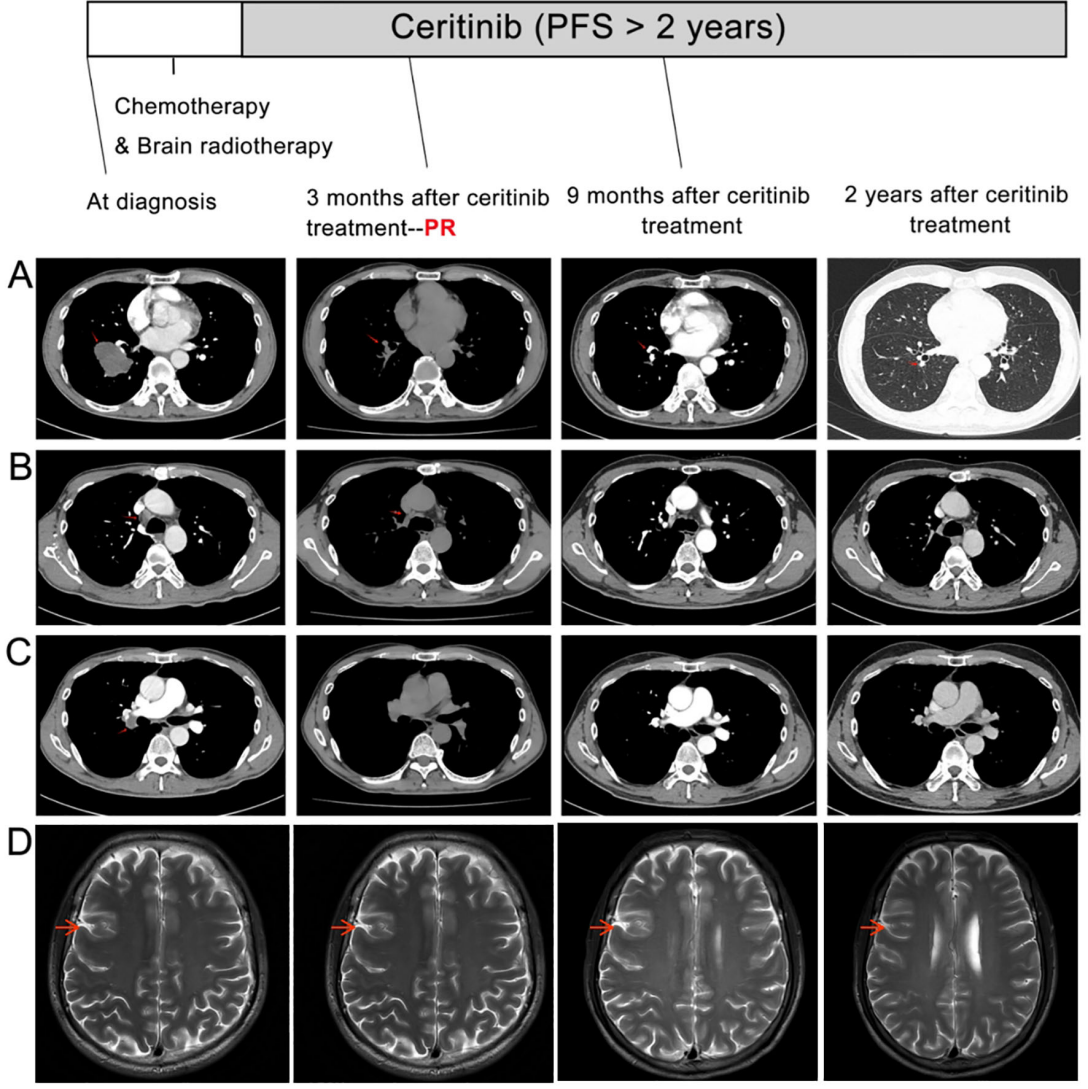
Treatment response on imaging. **(A-C)**: Baseline chest computed tomography (CT) scans at diagnosis showing **(A)** a 4.8 cm (longest diameter) ×3.5 cm mass in the right lower lobe (white arrow), **(B)** obstructive pneumonia, and **(C)** enlarged hilar/mediastinal lymph nodes (arrowheads). **(D)**: Baseline brain magnetic resonance imaging (MRI) revealing a metastatic lesion in the right frontal lobe (red arrow). Lower panels: Follow-up imaging at 3, 9, and 24 months after initiation of ceritinib (note: one cycle of platinum-based chemotherapy and whole-brain radiotherapy were administered between baseline imaging and the start of ceritinib) demonstrating **(A–C)** significant regression of the primary mass (2.4 cm × 1.2 cm at 3 months, yellow arrow), resolution of obstructive pneumonia, and reduced lymphadenopathy; and **(D)** complete resolution of the brain metastasis. RECIST assessment: The primary target lesion’s longest diameter decreased by 50% (from 4.8 cm to 2.4 cm), meeting partial response (PR) by RECIST 1.1 criteria (≥30% decrease in the sum of longest diameters of target lesions). The intracranial lesion met criteria for complete response on MRI.

**Figure 2:**
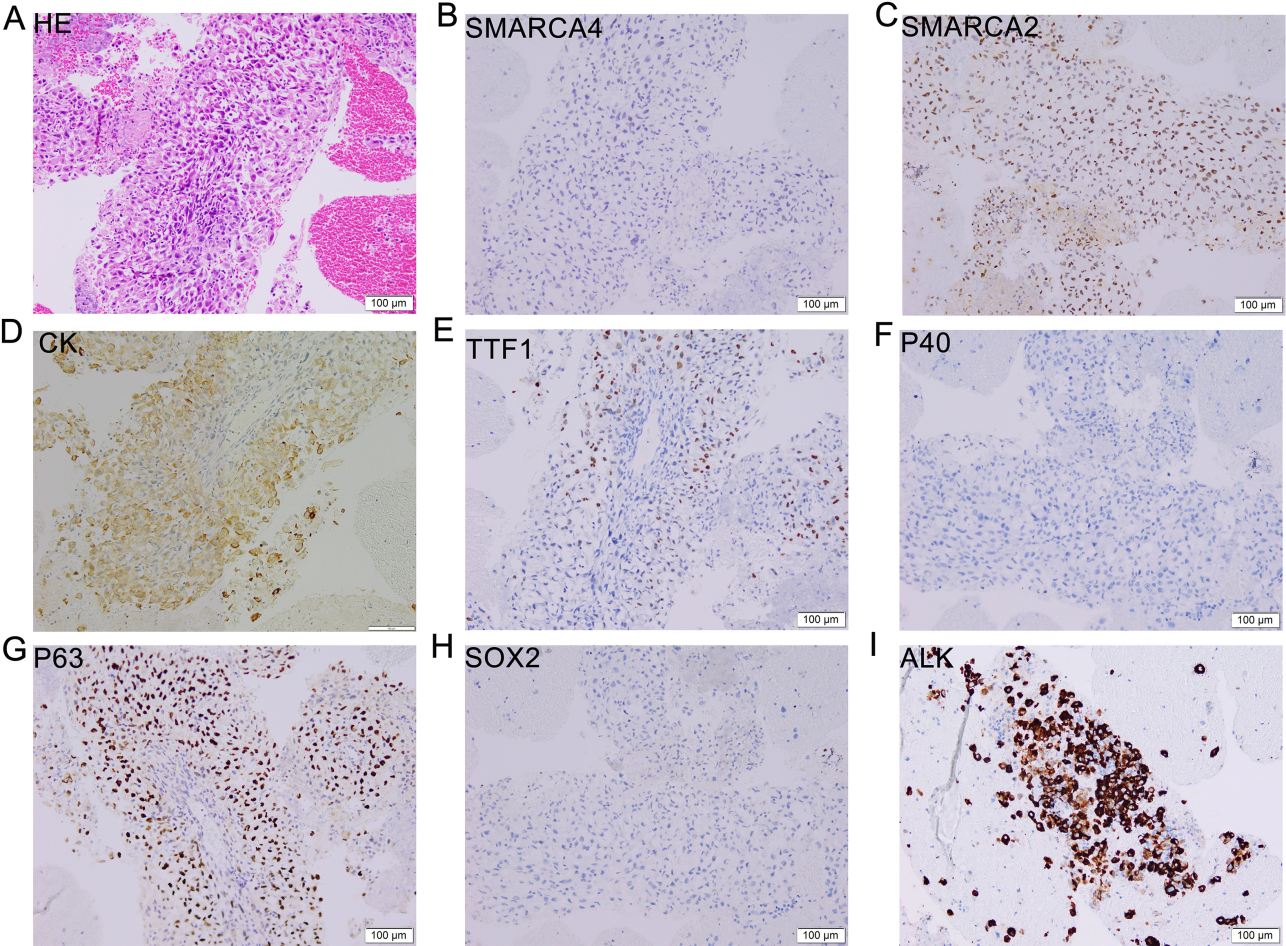
Histopathological and immunophenotypic features. Hematoxylin and eosin (H&E) staining **(A)** revealed poorly differentiated tumor morphology, while immunohistochemical analysis demonstrated weak positivity for P63 **(G)**, cytokeratin [CK, **(D)**], and TTF-1 **(E)**; and negative immunoreactivity for P40 **(F)**, SOX-2 **(H)**, SMARCA4 **(B)**, and SMARCA2 **(C)**, with Ventana IHC confirming positive ALK protein expression [**(I)**, D5F3 antibody] - consistent with SMARCA4-deficient NSCLC harboring ALK rearrangement. Scale bar: 100 μm; original image field = 750 μm × 750 μm; original magnification ×200.

A 51-year-old male with a 30-pack-year smoking history presented with chest pain, productive cough, and hemoptysis persisting for one month. Family history included laryngeal cancer (mother) and colorectal cancer (elder sister). The patient’s initial ECOG performance status was 1, with no comorbidities or concomitant medications. Aside from long-term smoking, he reported no alcohol consumption or occupational exposures related to lung cancer. Contrast-enhanced chest computed tomography (CT) revealed a 4.8 × 3.5 cm mass in the right lower lobe with associated hilar/mediastinal lymphadenopathy, obstructive pneumonia, and emphysema (**Figures 1A-C**). Brain MRI demonstrated a 1.2 cm metastatic lesion in the right frontal lobe (**Figure 1D**). These baseline CT and MRI scans were obtained prior to any treatment, including chemotherapy or radiotherapy. Bronchoscopic biopsy of the lung mass showed poorly differentiated carcinoma arranged in solid sheets and nest-like structures, with tumor cells displaying round-to-oval nuclei, occasional nucleoli, abundant cytoplasm, focal discohesion, and areas of necrosis. Immunohistochemistry (IHC) exhibited: Weak positivity (1+, <10% of tumor cells) for P63, cytokeratin (CK), and focal weak TTF-1; Positive Ki67 (proliferation index 30%); Negative staining for P40, Napsin A, ERG, CD31, SOX-2, SMARCA4, and SMARCA2 (**Figures 2A-H**). Both SMARCA4 and SMARCA2 exhibited complete nuclear loss in all tumor cells, with preserved nuclear expression in internal non-neoplastic elements, confirming adequate staining quality and true loss of BRG1/BRM expression. Ventana ALK IHC (D5F3) confirmed strong ALK protein expression (**Figure 2I**). PD-L1 IHC showed a tumor proportion score <1%. The epithelial morphology together with the epithelial marker profile supported the diagnosis of SMARCA4-deficient NSCLC, and SMARCA4-deficient thoracic sarcoma was excluded due to retained epithelial differentiation and absence of sarcomatoid features. The patient was staged as IV (cT2bN2M1) with synchronous brain metastasis.

**Figure 3:**
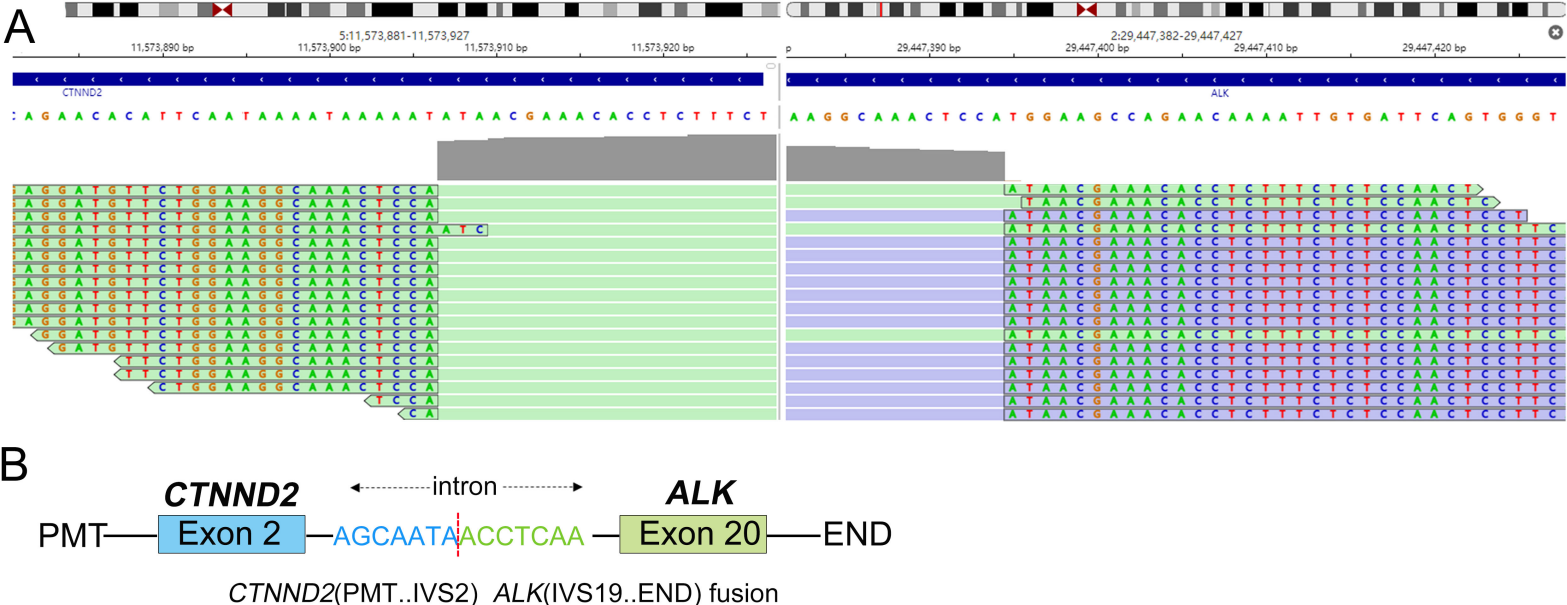
Molecular characterization of CTNND2-ALK fusion. **(A)** Integrative Genomics Viewer (IGV) visualization of next-generation sequencing reads demonstrating the CTNND2-ALK fusion junction. Arrows indicate the breakpoint between CTNND2 exon 2 and ALK exon 20. **(B)** Schematic representation of the novel CTNND2-ALK (exon 2:20) fusion protein structure: the N-terminal domain of CTNND2 is fused to the intact intracellular kinase domain of ALK.

Following diagnosis, the patient underwent one cycle of platinum-based chemotherapy (carboplatin AUC5 + pemetrexed 500mg/m²) and whole-brain radiotherapy (30 Gy/10 fractions). Capture-based next-generation sequencing (NGS) using a 1021-gene panel (Geneplus Technology) on FFPE tumor tissue revealed dual ALK fusions: CTNND2-ALK (exon 2:20) at 2.6% allele frequency, EML4-ALK (exon 13:20) at 5.2% allele frequency (**Table 1**; **Figures 3A, B**) and identified a coexisting TP53 p.R273L mutation. No other actionable alterations were detected. This was corroborated by strong ALK positivity on Ventana IHC (D5F3 clone, **Figure 2I**).

**Table 1:** Somatic mutations detected by next-generation sequencing, HGVS, Human Genome Variation Society.

Gene	Transcript	c.HGVS	p.HGVS	Allele frequency
Single-nucleotide variants and small insertions/deletions (gene)				
*ATR*	NM_001184.3	c.4093G>A	p.D1365N	1.6%
*C1S*	NM_001734.3	c.767C>T	p.P256L	11.9%
*CRLF2*	NM_022148.2	c.530C>A	p.A177D	11.0%
*LRP1B*	NM_018557.2	c.1920A>T	p.L640F	7.6%
*TP53*	NM_000546.5	c.818G>T	p.R273L	9.5%
Fusions (gene)				
*CTNND2-ALK*	NM_001332.2; NM_004304.4 (Functional region: EX2:EX20)			2.6%
*EML4-ALK*	NM_019063.3; NM_004304.4 (Functional region: EX13:EX20)			5.2%

HGVS, Human Genome Variation Society.

Given the presence of dual ALK rearrangements with synchronous brain metastasis and the proven systemic and intracranial efficacy of ceritinib demonstrated in the ASCEND-4 trial (8), first-line ceritinib (750 mg daily, as used in ASCEND-4) was initiated. At 3-month assessment, chest CT showed 75% reduction in the primary lesion (2.4×1.2 cm vs. baseline 4.8×3.5 cm), resolved obstructive pneumonia, and regressed lymphadenopathy (**Figures 1A-C**, lower panels). Brain Magnetic Resonance Imaging (MRI) confirmed complete remission of the metastatic lesion (**Figure 1D**). RECIST 1.1 criteria defined partial response (PR: target lesion sum diameter decrease >30%). Continued ceritinib treatment resulted in further regression of the primary mass to 1.1 cm at 9 months. During follow-up, the patient’s symptoms gradually improved, and his ECOG performance status returned to 0. At last follow-up (24+months), the patient maintains progression-free survival with ongoing treatment response and no grade ≥2 adverse events (CTCAE v5.0 criteria).

## Discussion

We report the first documented case of SMARCA4-deficient lung adenocarcinoma harboring dual ALK fusions (CTNND2-ALK/EML4-ALK) with sustained response to ceritinib, providing critical evidence for ALK-targeted therapy in this aggressive subtype.

SMARCA4-dNSCLC diagnosis relies on BRG1 protein loss by IHC or SMARCA4 mutations by NGS, though truncating mutations drive BRG1 deficiency more consistently than missense variants (81% vs. 0% loss rates) (5, 9). Our case showed BRG1 loss without detectable SMARCA4 mutations, potentially due to non-mutational mechanisms or NGS panel limitations (10). Genomically, SMARCA4-dNSCLC typically harbors TP53/KRAS/STK11/KEAP1 alterations but rarely actionable fusions (9). Prior ALK-rearranged SDTT reports involved only SMARCA4-UT (not NSCLC) responding to alectinib (9), while our TP53-mutated case represents true SMARCA4-dNSCLC. The co-occurrence of SMARCA4 deficiency with two distinct ALK rearrangements is biologically uncommon, potentially reflecting the heightened genomic instability associated with SWI/SNF complex dysfunction (11). Despite the aggressive phenotype typically attributed to SMARCA4 loss, the patient exhibited a profound and durable response to ceritinib, indicating that ALK signaling remained the dominant oncogenic driver in this tumor (12).

Notably, CTNND2-ALK (exon2:20) is a novel fusion retaining ALK’s kinase domain. CTNND2 (5p15) encodes an adhesion protein overexpressed in cancers (13), potentially enhancing oncogenicity when fused to ALK. Although current NCCN guidelines preferentially recommend alectinib or brigatinib for first-line therapy in ALK-positive NSCLC, ceritinib remains an evidence-based and guideline-supported option with demonstrated systemic and intracranial activity. In this case, treatment selection was made based on the robust efficacy signals from ASCEND-4, the patient’s clinical status with symptomatic brain metastasis, and institutional therapeutic considerations at the time of decision-making. Therefore, ceritinib was chosen as a clinically appropriate and guideline-supported first-line therapy (8).

Although complex ALK rearrangements may confer resistance (9, 13), our patient achieved 24-month PFS with ceritinib. We hypothesize that: (i) the preserved ALK kinase domain in both fusions maintains ceritinib binding affinity; (ii) concurrent radiotherapy may have eliminated resistant subclones; and (iii) the EML4-ALK (E13:A20) variant has high intrinsic TKI sensitivity (14, 15).

Although the patient received one cycle of platinum-pemetrexed chemotherapy and whole-brain radiotherapy (WBRT) before the NGS results became available, we acknowledge that these induction treatments may introduce some confounding effects when interpreting the early radiologic response. Nevertheless, several considerations suggest that their overall influence on long-term outcomes was limited. First, a single cycle of platinum-based chemotherapy typically produces modest and short-lived responses in ALK-positive NSCLC and would not be expected to account for the profound and durable tumor shrinkage observed over more than 24 months. Second, WBRT can explain the remission of the intracranial lesion but cannot account for the marked and continued regression of the thoracic disease. Third, the depth, speed, and persistence of response-including further tumor shrinkage beyond the initial three months-are highly characteristic of ALK-TKI-driven therapeutic benefit rather than cytotoxic chemotherapy. Nonetheless, the absence of intermediate imaging between induction therapy and ceritinib initiation remains a limitation.

The observed discordance between the low DNA VAF and strong ALK protein expression likely reflects sampling heterogeneity and the greater biological relevance of RNA or protein-level expression for ALK-driven oncogenesis. Although RNA-based NGS or FISH would provide ideal orthogonal validation, these assays were not performed due to patient-related constraints, and this has been acknowledged as a limitation.

This case establishes ceritinib’s efficacy in ALK-rearranged SMARCA4-dNSCLC and underscores NGS’s vital role in identifying targetable drivers in molecularly complex tumors.

## Data Availability

The raw data supporting the conclusions of this article will be made available by the authors, without undue reservation.
